# Pulmonary drug delivery and retention: A computational study to identify plausible parameters based on a coupled airway-mucus flow model

**DOI:** 10.1371/journal.pcbi.1010143

**Published:** 2022-06-02

**Authors:** Aranyak Chakravarty, Mahesh V. Panchagnula, Alladi Mohan, Neelesh A. Patankar

**Affiliations:** 1 School of Nuclear Studies and Application, Jadavpur University, Kolkata, India; 2 Department of Applied Mechanics, Indian Institute of Technology Madras, Chennai, India; 3 Department of Medicine, Sri Venkateswara Institute of Medical Sciences, Tirupati, India; 4 Department of Mechanical Engineering, Northwestern University, Evanston, Illinois, United States of America; Stanford University, UNITED STATES

## Abstract

Pulmonary drug delivery systems rely on inhalation of drug-laden aerosols produced from aerosol generators such as inhalers, nebulizers etc. On deposition, the drug molecules diffuse in the mucus layer and are also subjected to mucociliary advection which transports the drugs away from the initial deposition site. The availability of the drug at a particular region of the lung is, thus, determined by a balance between these two phenomena. A mathematical analysis of drug deposition and retention in the lungs is developed through a coupled mathematical model of aerosol transport in air as well as drug molecule transport in the mucus layer. The mathematical model is solved computationally to identify suitable conditions for the transport of drug-laden aerosols to the deep lungs. This study identifies the conditions conducive for delivering drugs to the deep lungs which is crucial for achieving systemic drug delivery. The effect of different parameters on drug retention is also characterized for various regions of the lungs, which is important in determining the availability of the inhaled drugs at a target location. Our analysis confirms that drug delivery efficacy remains highest for aerosols in the size range of 1-5 *μ*m. Moreover, it is observed that amount of drugs deposited in the deep lung increases by a factor of 2 when the breathing time period is doubled, with respect to normal breathing, suggesting breath control as a means to increase the efficacy of drug delivery to the deep lung. A higher efficacy also reduces the drug load required to be inhaled to produce the same health effects and hence, can help in minimizing the side effects of a drug.

## Author summary

Pulmonary drug delivery systems utilize the respiratory mechanism to directly deliver drugs to a target region of the lungs. The drug molecules deposit in the mucus lining, on reaching the target region, and are simultaneously transported away from the target region due to mucociliary transport and molecular diffusion. The availability of drugs at a target lung region and hence, efficacy of the drugs, therefore, determined by the delivery and retention of the drugs at the target region. The present study computationally solves the coupled transport equations to identify the conditions conducive for drug delivery and retention in the deep lungs. Drug delivery efficacy to the deep lung is observed to be highest for 1–5 *μ*m aerosols. Breathing time period is also observed to influence efficacy. The amount of drugs deposited in the deep lung is observed to increase by a factor of 2 when the breathing time period is doubled with respect to normal breathing period. Such insights gained from this analysis will potentially help in devising mechanisms for increasing drug availability in the deep lung which is essential in achieving systemic drug delivery.

This is a *PLOS Computational Biology* Methods paper.

## Introduction

The lung is one of the most exposed organs of the human body [1]. The dichotomous branching structure of the lung—starting from the trachea and culminating in the alveolar sacs—provides a mechanism by which air from the surrounding atmosphere is drawn into the lungs during inhalation and expired out during exhalation. Pulmonary drug delivery systems take advantage of the respiration process to deliver drug molecules to the lung through inhalation. The drug molecules may be in the form of dry powders or liquid aerosols, and are administered in a non-invasive manner with the help of aerosol generators such as inhalers, nebulisers etc. [2, 3]. Once inhaled, the powdered/aerosolised drugs are transported along the respiratory tract where they deposit depending on their physio-chemical properties as well as breathing characteristics and physiological conditions. Thus, drugs can be delivered locally to a targeted region of the lung for treatment of respiratory diseases, such as asthma or COPD [3]. Such targeted delivery can potentially lead to smaller overall drug dose and reduced side effects. Systemic drug delivery can also be achieved by targeting delivery to the alveolar region of the lung where the drugs can be easily absorbed into the systemic blood circulation through the thin blood-gas barrier and the large alveolar surface area [1].

The transport of the inhaled aerosols within the respiratory tract is governed by the combined effects of unsteady convective air flow, gravitational settling, and aerosol diffusion in air [4, 5]. At the same time, the inhaled aerosols are deposited primarily due to diffusion, sedimentation, and inertial impaction [4–7], which depend significantly on aerosol properties and other physiological parameters [4]. It has been observed that a major portion of the inhaled aerosols are deposited in the naso-pharyngeal region of the upper respiratory tract [4, 5]. Basu et al. [8] identified that aerosols in the range of 2–20 *μ*m are ideal for nasopharyngeal deposition. The study also observed that a significant portion of 5 *μ*m and smaller aerosols may avoid deposition in the nasopharyngeal region and escape to the lower respiratory tract during inhalation. These aerosols may again deposit in various regions of the lower respiratory tract before reaching the target region. This effectively reduces the actual dose reaching the target region of the lung. For example, aerosols larger than 10 *μ*m have been observed to be completely deposited in the upper airways of the lower respiratory tract and do not reach the alveolar region at all [9, 10]. The physio-chemical properties (size, shape, morphology, chemical composition etc.) of the inhaled aerosol must, as such, be tailored to facilitate drug delivery to the target region depending on breathing characteristics and other physiological conditions.

The inhaled aerosols, containing the drug molecules, are deposited in the respiratory mucus lining the inner surface of the lower respiratory tract [11–13]. The mucus layer forms the upper sub-layer of the airway surface liquid that remain in contact with the airway lumen and lies above the periciliary layer which remains in contact with the epithelial cells. The airway surface liquid and the mucus layer in particular, thus, prevents the deposited drug molecules from coming in direct contact with the epithelial cells (which lie underneath the mucus lining) and the capillaries (which remain beyond the epithelium) [11]. The respiratory mucus, therefore, acts as a barrier to drug absorption. In addition, the epithelial cells are also lined with cilia which beat metachronously within the periciliary layer [12, 14] transporting the mucus, and the deposited drug molecules, from the distal airways towards the pharyngeal region. Mucociliary clearance, as such, further prevents effective absorption of the deposited drug molecules. It is, therefore, essential to consider mucociliary transport while studying drug delivery in the lungs. However, mathematical models published in the literature have not accounted for mucociliary transport while investigating pulmonary drug delivery in the lower respiratory tract.

Thus, in order to computationally explore the pulmonary drug delivery mechanism, one needs a mathematical model that takes into account aerosol transport (in airways) and drug molecule transport (in mucus), since these transport processes occur simultaneously within the lung. Although such coupled models have been used in the recent past to study aerosol transport in the nasal passage [15, 16], such a model is being reported for the first time for investigating the fate of drug-laden aerosols within the lower respiratory tract, especially using an approach that is mathematically rigorous.

Different techniques (Eulerian, Lagrangian and combinations thereof) have been used in the past to computationally model aerosol transport and deposition [4] in specific regions of the respiratory tract [17–20] as well as the whole lung [21, 22]. Here, *whole lung* models consider the lungs to be a network of interconnected branching channels with varying dimensions based on lung morphometry. The computational model used in the present analysis is based on one such *whole lung* model [9, 21]—based on a Weibel [23] lung geometry with appropriate modifications. The primary goal is to use this mathematical model to identify situations that can lead to the transport of aerosols, containing the drug molecules, from the pharyngeal region to the deep lungs. The model is also used to determine the conditions that promote retention of the deposited drug molecules in the lungs and thereby, increase the bioavailability of the drugs.

Although the mathematical model has been used here to specifically study drug delivery to the lungs, the same model can be utilised to study other similar physical processes involving exposure of the lungs to foreign particles such as pollutant (smoke, dust etc.) and pathogen (virus, bacteria etc.) deposition and clearance from the lungs.

## Methods

### Idealisation of the lung geometry

**Fig 1:**
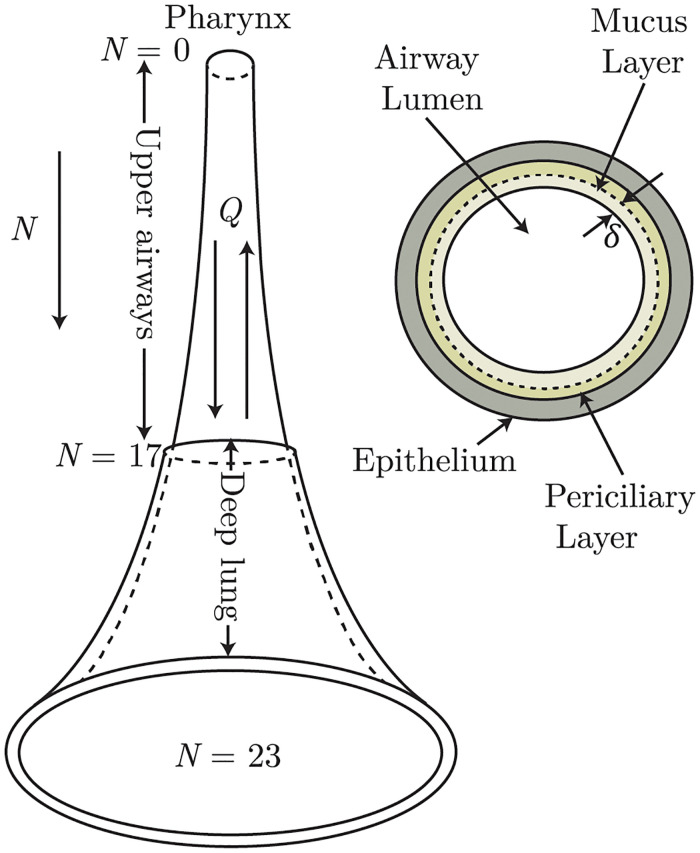
Schematic illustration of the one-dimensional *trumpet* model that is used in the present analysis to approximate the dichotomous network structure of a human lungs. A cross-sectional view of a single airway branch is also shown to illustrate the arrangement of the airway lumen and the epithelial lining with respect to the intermediate mucus layer and the periciliary layer.

The physiological dichotomous branching network of human lungs is approximated in this work by a one-dimensional *trumpet* model (Fig 1). While this model cannot account for the effects of heterogeneity in the lungs, it is still a tractable model for the whole lungs in order to capture key trends.

The airway is modeled as a continuous one-dimensional channel of variable cross-sectional area, where the length is divided into 24 generations (*N* = 0–23; *N* is the generation number), based on morphometric data of a human lungs [23]. For a dichotomous tree, the number of bronchioles in each generation is 2^*N*^, while the length (*L*) and the total cross-sectional area (*A*) at each generation is calculated using a power-law function as

$$\begin{array}{c} L\left(N\right)=L_{0}\alpha^{N},A\left(N\right)=A_{0}{\left(2\beta \right)}^{N}, \end{array}$$ (1)

where *L*_0_ and *A*_0_ are the length and cross-sectional area at *N* = 0, respectively (see *Table A in* S1 Text for magnitudes). The length-change (*α*) and area-change (*β*) factors are selected (*Table A in* S1 Text) such that the computed length and area at each generation matches Weibel’s morphometric data [23]. Although *N* is an integer, it is treated as a continuous variable in all transport equations for computational convenience. The airway length (*x*), in terms of the lung generation number *N*, is given by

$$\begin{array}{c} x\left(N\right)=\frac{L_{0}\left(1-\alpha^{N+1}\right)}{1-\alpha }. \end{array}$$ (2)

Alveolation of the distal lung airways is considered *N* = 18 onwards, consistent with human lungs [23], by considering additional surface area in the relevant generations (see *Table B in* S1 Text). The corresponding lung generations are referred to as the *deep lung*, while the rest of the proximal airways of the lower respiratory tract are referred to as *upper airways* (see Fig 1).

The modeled system of airways and alveoli is also assumed to be lined by a thin mucus layer separating the airway lumen from the underlying periciliary layer and the epithelium (see Fig 1). The periciliary layer, the epithelium and the ciliary motion driving mucus transport are not explicitly modelled. Instead, mucociliary transport is accounted for by assuming a convective motion in the mucus layer from the deeper generations towards the 0^*th*^ generation. The thickness (*δ*), the total cross-sectional area of the mucus layer (*A*_*m*_), and the convective mucus velocity (*V*_*m*_) at different lung generations are estimated as

$$\begin{array}{c} \begin{array}{cc} \delta \left(N\right)=\delta_{0}\zeta^{N},\; & A_{m}\left(N\right)=A_{m,0}{\left(2\sqrt{\beta }\zeta \right)}^{N},\\ V_{m}\left(N\right) & =V_{m,0}\varepsilon^{N}\text{,}\;\text{for}\;\text{N}<18,\\ & =0\text{,}\;\text{for}\;\text{N}\geq 18, \end{array} \end{array}$$ (3)

where *δ*_0_, *A*_*m*,0_, and *V*_*m*,0_ are the mucus thickness, area, and velocity at *N* = 0, respectively (see *Table A in* S1 Text). The magnitudes of the change factors *ζ* and *ε* (see *Table A in* S1 Text) are chosen based on experimental data [12]. *V*_*m*_ is zero beyond *N* = 18 (Eq 3) due to the absence of appreciable mucociliary transport in the deep lungs [11]. *δ* and *V*_*m*_ are also assumed to be temporally invariant in this analysis. [12].

### Aerosol transport in airways

The one-dimensional transport equation for aerosols in the idealized lung geometry is

$$\begin{array}{c} \frac{\partial (Ac_{a})}{\partial t}+H\frac{\partial (Qc_{a})}{\partial N}=H\frac{\partial }{\partial N}(AD_{a}H\frac{\partial c_{a}}{\partial N})-L_{D}c_{a}, \end{array}$$ (4)

where *c*_*a*_, *Q*, and *D*_*a*_ are aerosol concentration, volume flow rate of air during breathing, and aerosol diffusivity in air, respectively, and $H\left(N\right)=\frac{\partial N}{\partial x}$. The coefficient *L*_*D*_ models aerosol deposition in the airway mucus. Eq 4 assumes that the aerosols are monodispersed, do not coagulate, and do not affect the airflow in the lungs. Consistent with the focus of this study, it is assumed that the only source of aerosols is at the entrance to the 0^*th*^ generation, presumably from an aerosol generator. No additional aerosolization of the mucus or aerosol source are considered within the lungs. The inhaled aerosols are either deposited or washed out of the airways. Eq 4 is reduced to a dimensionless form (Eq 6) using scalings defined in Eq 5 below (see S1 Text)

$$\begin{array}{c} \begin{array}{cc} \tau = & \frac{t}{T_{b}},\phi_{a}=\frac{c_{a}}{c_{a,0}},T_{a}=\frac{L_{0}A_{0}}{|Q_{max}|},St_{a}=\frac{T_{a}}{T_{b}},\\ & Pe_{a}=\frac{|Q_{max}|L_{0}}{A_{0}D_{a}},D_{a}=\frac{k_{B}TC_{S}}{3\pi \mu_{a}d_{a}}. \end{array} \end{array}$$ (5)

$$\begin{array}{c} \begin{array}{cc} Pe_{a}St_{a}{\left(2\alpha \beta \right)}^{N} & \frac{\partial (\phi_{a})}{\partial \tau }=\frac{\partial }{\partial N}[({\left(\frac{2\beta }{\alpha }\right)}^{N}{\left(\frac{1-\alpha }{\alpha (\text{ln}\alpha )}\right)}^{2}\frac{\partial \phi_{a}}{\partial N})\\ & +(Pe_{a}q\left(t\right)(\frac{1-\alpha }{\alpha \;\text{ln}(\alpha )})\phi_{a})]-L_{D}^{\prime }\phi_{a}, \end{array} \end{array}$$ (6)

where *Pe*_*a*_, *St*_*a*_, *ϕ*_*a*_, and *τ* represent aerosol Peclet number, airway Strouhal number, dimensionless aerosol concentration, and dimensionless time, respectively. Note that *Pe*_*a*_ refers to the aerosol Peclet number at *N* = 0 only. As such, even if *Pe*_*a*_ is extremely large, the local Peclet numbers at the higher generations can remain small. *T*_*a*_ is the convective airflow timescale and *T*_*b*_ is the breathing time period. *D*_*a*_ is calculated using the Stokes-Einstein relation, where *k*_*B*_, *T*, *C*_*S*_, *μ*_*a*_, and *d*_*a*_ are the Boltzmann constant, temperature, Cunningham slip correction factor, viscosity of air, and aerosol diameter, respectively [17]. $L_{D}^{\prime }$ is the dimensionless aerosol deposition coefficient which is determined using empirical models for various deposition mechanisms (see S1 Text).

*q*(*t*) in Eq 6 is a sinusoidal function accounting for airflow variation during breathing (*Q* = *Q*_*max*_ *q*(*t*)). Analysis shows that Womersley number ($Wo=d\sqrt{\frac{\rho \omega }{\mu }}$), which is used to quantify pulsatile flows such as airflow in the lungs during breathing, remains in the range of 6–0.1 in the respiratory tract. It is observed that *Wo* < 2 when *N* > 2 implying that unsteady effects decrease as one goes deeper inside the respiratory tract. Also, $Wo∼\sqrt{\omega }$ suggests that a longer breath (smaller pulsatile frequency) would further reduce the magnitude of *Wo* and minimize the unsteady effects. This eliminates the need for solving separate airflow equations.

### Drug molecule transport in mucus

The one-dimensional transport equation for the deposited drug molecules in the mucus is formulated considering mucociliary transport and diffusion of the deposited drug molecules in the mucus. It is expressed as

$$\begin{array}{c} \frac{\partial (A_{m}c_{d})}{\partial t}+H\frac{\partial (Q_{m}c_{d})}{\partial N}=H\frac{\partial }{\partial N}(A_{m}D_{d}H\frac{\partial c_{d}}{\partial N})+L_{D}c_{a}\phi_{l}, \end{array}$$ (7)

where *c*_*d*_, *Q*_*m*_, and *D*_*d*_ are drug concentration in the mucus, volume flow rate of mucociliary transport, and drug molecule diffusivity in the mucus, respectively. *ϕ*_*l*_ is the drug load in the droplets, defined as the quantity of drug molecules contained per unit quantity of droplets. The term *L*_*D*_ *c*_*a*_ *ϕ*_*l*_ takes into account the drug molecules being introduced into the mucus due to aerosol deposition. Further absorption of the deposited drugs across the epithelium into the blood stream is not considered presently. Eq 7 is converted to a dimensionless form (Eq 9) using scalings defined in Eq 8 below (see S1 Text)

$$\begin{array}{c} \begin{array}{cc} \tau = & \frac{t}{T_{b}},\phi_{d}=\frac{c_{d}}{c_{d,0}},c_{d,0}=\phi_{l}c_{d,0}\frac{A_{0}}{A_{m,0}},T_{m}=\frac{L_{0}}{|V_{m,0}|},\\ & St_{m}=\frac{T_{m}}{T_{b}},Pe_{d}=\frac{|V_{m,0}|L_{0}}{D_{d}},D_{d}=\frac{k_{B}T}{3\pi \mu_{m}d_{d}}. \end{array} \end{array}$$ (8)

$$\begin{array}{c} \begin{array}{cc} Pe_{d}{\left(2\alpha \zeta \sqrt{\beta }\right)}^{N} & St_{m}\frac{\partial \phi_{d}}{\partial \tau }=\\ & \frac{\partial }{\partial N}[({\left(\frac{2\zeta \sqrt{\beta }}{\alpha }\right)}^{N}{\left(\frac{1-\alpha }{\alpha \;\text{ln}(\alpha )}\right)}^{2}\frac{\partial \phi_{d}}{\partial N})\\ & -(Pe_{d}{\left(2\varepsilon \zeta \sqrt{\beta }\right)}^{N}\phi_{d}))]+(L_{D}^{\prime }\frac{D_{a}}{D_{d}}\phi_{a}), \end{array} \end{array}$$ (9)

where *ϕ*_*d*_, *Pe*_*d*_, and *St*_*m*_ are the dimensionless drug concentration, drug Peclet number, and mucus layer Strouhal number, respectively. Also note that *Pe*_*d*_ refers to the drug molecule Peclet number at *N* = 0 only. *T*_*m*_ denotes the time-scale for mucociliary transport. *D*_*d*_ is estimated using the Stokes-Einstein relation, where *μ*_*m*_ and *d*_*d*_ are the viscosity of the mucus and the drug molecule diameter, respectively. The last term on the right hand side of Eq 9 is the dimensionless drug source due to aerosol deposition.

### Initial and boundary conditions

The lungs are assumed to be initially devoid of aerosols and drugs, i.e., *ϕ*_*a*_|_*τ* = 0_ = *ϕ*_*d*_|_*τ* = 0_ = 0 at all generations. It is also assumed that *N* = 0 of the lungs is exposed to drug-laden aerosols, presumably from an aerosol generator, for a specific exposure duration (*τ*_*exp*_). The aerosols are breathed in during inhalation (Eq 10) and washed out during exhalation (Eq 11). In contrast, the drugs are always assumed to be washed out of *N* = 0, along with the mucus, irrespective of inhalation/exhalation (Eq 12). At the distal end of the lungs (*N* = 23), the total advection-diffusion flux of both aerosols and drugs is assumed to be zero (Eq 13). Mathematically, these conditions are expressed as follows

$$\begin{array}{c} \begin{array}{cc} \phi_{a}{|}_{N=0} & =1,\tau \leq \tau_{exp},\\ & =0,\tau >\tau_{exp}, \end{array} \end{array}$$ (10)

$$\begin{array}{c} \frac{\partial (F_{a})}{\partial N}{|}_{N=0}=0,\tau >0, \end{array}$$ (11)

$$\begin{array}{c} \frac{\partial (F_{d})}{\partial N}{|}_{N=0}=0,\tau >0, \end{array}$$ (12)

$$\begin{array}{c} F_{a}{|}_{N=23}=F_{d}{|}_{N=23}=0,\tau >0, \end{array}$$ (13)

where *F*_*a*_ and *F*_*d*_ are the total advection-diffusion flux in the aerosol transport (Eq 6) and drugs transport equation (Eq 9), respectively (see S1 Text). Detailed derivation of the mathematical model and its validation (*Fig B in* S1 Text) are provided in S1 Text.

## Results and discussion

**Fig 2:**
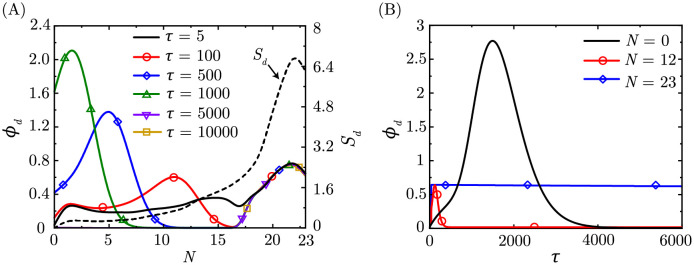
(A) Aerosol deposition (*S*_*d*_ $=\int \int L_{D}^{\prime }\phi_{d}d\forall d\tau$) within the lungs at the end of exposure and drug concentration (*ϕ*_*d*_) within the lungs at different time instances (*τ*) (B) Temporal change in *ϕ*_*d*_ at *N* = 0, 12, 23. The results are shown for *Pe*_*a*_ = 2.85 × 10^10^, *St*_*a*_ = 0.0095, *Pe*_*d*_ = 4.56 × 10^7^, *St*_*m*_ = 359.7122, *τ*_*exp*_ = 5.

Drug-laden aerosols are deposited in the respiratory mucus primarily during inhalation. The deposited drug molecules diffuse in the mucus layer and are transported upstream (towards the mouth) via mucociliary advection. To obtain the key deposition and washout trends, simulations were done assuming that drug-laden aerosols are entering the lungs for five breaths, i.e., exposure time *τ*_*exp*_ = 5. Extrapolation to longer exposure times and its impact on drug retention will be discussed separately. It is seen that the (scaled) drug concentration in the mucus (*ϕ*_*d*_), at the end of the exposure duration (*τ* = 5), qualitatively follows aerosol deposition *S*_*d*_ ($=\int \int L_{D}^{\prime }\phi_{a}d\forall d\tau$; see Fig 2A).

Drug molecules deposited in the conducting airways (*N* < 18; *N* represents the lung generation) is transported upstream towards the mouth (*N* = 0). This results in higher drug concentration *ϕ*_*d*_ in the upper airways (lower *N*) primarily due to smaller mucus volume. Eventually, the drugs are washed out of the lungs (see Fig 2A). The temporal change in *ϕ*_*d*_ at the mouth (Fig 2B) also corroborates this conclusion.

In contrast, drugs deposited in the deeper generations (*N* ≥ 18) are not subjected to mucociliary transport. Therefore, *ϕ*_*d*_ undergoes a gradual change due to weak diffusive transport. As such, drugs deposited in the deep lungs persist for a much longer time as compared to that deposited in the upper airways. This is also clearly evident from Fig 2.

Deep lung (alveolar) deposition of the drugs is beneficial for systemic drug delivery primarily due to the thin mucus layer in the deep lung and the large surface area of the alveoli and the alveolated bronchioles in contact with the blood vessels. This enables the deposited drugs to come in close contact with the blood vessels and increases the probability of the drugs entering the blood stream, thereby ensuring systemic drug delivery. A longer residence time of the deposited drugs within the deep lungs further increases the probability of systemic drug delivery. Thus, it is important to understand the various effects that cause the drugs to deposit and persist in the deep lungs. This is discussed next. Physiologically relevant ranges are chosen for all parameters in this study (see *Tables A* and *C* in S1 Text for more details).

### Effect of aerosol size on drug deposition in the deep lungs

**Fig 3:**
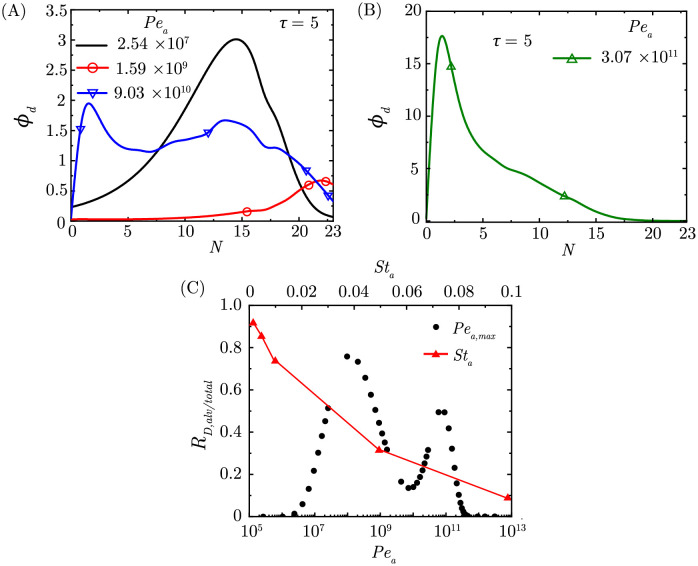
(A-B) *ϕ*_*d*_ within the lungs for different *Pe*_*a*_ at the end of exposure (*St*_*a*_ = 0.0095, *Pe*_*d*_ = 4.56 × 10^7^, *St*_*m*_ = 359.7122, *τ*_*exp*_ = 5) (C) Change in fraction of droplets deposited in the deep lungs to that in the whole lungs (*R*_*D*,*alv*/*tot*_) with variation in *Pe*_*a*_ and *St*_*a*_.

Aerosol Peclet number (*Pe*_*a*_) is defined as the ratio of advective transport to diffusive transport of aerosols in air (see Eq 5). Greater peak inspiratory flow rate will lead to larger *Pe*_*a*_, which implies greater advective transport. Smaller aerosols exhibit greater diffusive transport leading to smaller *Pe*_*a*_. Intuitively, one would expect the aerosols to reach deeper parts of the lungs at larger *Pe*_*a*_ due to stronger advective transport in air. However, deposition trends are non-monotonic (see Fig 3A and 3B). Specifically, deposition in the deep lungs increases up to *Pe*_*a*_ = 1.59 × 10^9^ and then decreases. Additionally, the peak of *ϕ*_*d*_ is observed in lower generations (*N* < 18) at both small and large values of *Pe*_*a*_. This is because, at small *Pe*_*a*_, the advection is not strong enough to carry the aerosols into the deep lungs, whereas at large *Pe*_*a*_ the aerosols deposit in the upper airways due to the impaction mechanism (see Fig 3B). Drug retention within the lungs, however, remains unaffected when *Pe*_*a*_ is changed, since it affects neither mucociliary transport nor drug diffusivity in mucus (see *Fig C in* S1 Text for more details).

Fig 3C shows the fraction of the drug-laden aerosols deposited in the deep lungs at different values of *Pe*_*a*_. It is seen that deposition of the aerosols in the deep lungs occurs when 2.37 × 10^6^ < *Pe*_*a*_ < 3.07 × 10^11^. This range translates to aerosol diameters of 10 *μ*m to 0.003 *μ*m for normal breathing in a healthy individual (tidal volume of 1000 ml and *T*_*b*_ = 4 s). Within this range, deposition is comparatively less for 4.29 × 10^9^ < *Pe*_*d*_ < 1.6 × 10^10^ (aerosols diameters ∼0.2–0.6 *μ*m).

In summary, aerosols smaller than 10 *μ*m diameter will tend to deposit in the deep lungs under normal breathing conditions. A survey of the literature reveals a wide variation in the size of the aerosols (0.1–100 *μ*m) produced from commercially available aerosol generators (inhalers, nebulizers etc.) [3]. Basu et al. [24] reported a mass median diameter larger than 40 *μ*m, while Kooji et al. [2] reported the mass median diameter of aerosols produced from various ultrasonic nebulizers to be in the range of 1–10 *μ*m although larger droplets (∼50 *μ*m) were also observed. It is, thus, evident that there needs to be focused investigations on rethinking the design of such aerosol generators for achieving better drug delivery to the deep lungs.

### Effect of mucus advection and viscosity on drug retention

**Fig 4:**
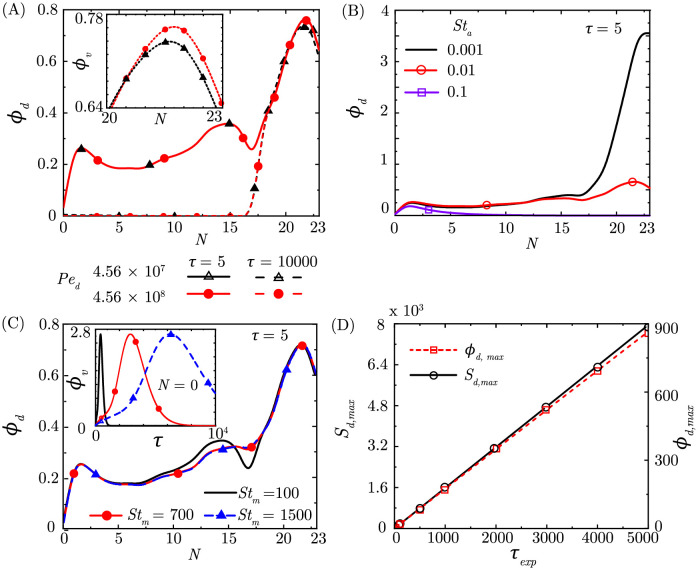
(A) *ϕ*_*d*_ within the lungs at the end of exposure (*τ* = 5) and at *τ* = 10000 for two different *Pe*_*d*_ (*Pe*_*a*_ = 2.85 × 10^10^, *St*_*a*_ = 0.0095, *St*_*m*_ = 359.7122, *τ*_*exp*_ = 5). A zoomed view of *ϕ*_*d*_ in the deep lungs is shown as inset to adequately highlight the difference in *ϕ*_*d*_ for the two cases (B) *ϕ*_*d*_ within the lungs for different *St*_*a*_ at the end of exposure to drug-laden aerosols (*Pe*_*a*_ = 2.85 × 10^10^, *Pe*_*d*_ = 4.56 × 10^7^, *St*_*m*_ = 359.7122, *τ*_*exp*_ = 5) (C) *ϕ*_*d*_ within the lungs at the end of exposure for various *St*_*m*_ at *τ* = 5 (*Pe*_*a*_ = 2.85 × 10^10^, *Pe*_*d*_ = 4.56 × 10^7^, *St*_*a*_ = 0.0095, *τ*_*exp*_ = 5). The temporal change of *ϕ*_*d*_ at *N* = 0 is shown as inset to highlight faster drug washout from the upper airways at smaller *St*_*m*_. (D) Increase in aerosol deposition (*S*_*d*_) and *ϕ*_*d*_ within the lungs with rise in exposure time (*Pe*_*a*_ = 2.85 × 10^10^, *Pe*_*d*_ = 4.56 × 10^7^, *St*_*a*_ = 0.0095, *St*_*m*_ = 359.7122).

Drug Peclet number (*Pe*_*d*_) is the ratio of advective mucociliary transport and diffusive transport of the drug molecules in the mucus layer (see Eq 8). An increase in *Pe*_*d*_ indicates a larger contribution of mucociliary transport (or a smaller impact of diffusion) in the overall transport process and vice-versa. The typical range of *Pe*_*d*_ in humans is such that advection dominates and there are no significant alterations to drug transport in the upper airways (see Fig 4A). However, in the deep lungs, where there is no mucociliary advection, drug retention is enhanced at a larger *Pe*_*d*_ (defined based on upper airway parameters) due to comparatively smaller diffusion (see Fig 4A inset).

Drug molecule diffusivity (*D*_*d*_) depends inversely on the drug molecule size and viscosity of the mucus (see Eq 8). A smaller molecule and lower viscosity of the mucus would, therefore, inhibit drug retention in the deep lungs but would not significantly alter drug retention in the upper airways due to weak dependence on *Pe*_*d*_. Controlling the size of the drug molecule and mucus property modification is therapeutically viable and can be a possible approach to enhance drug retention in the deep lungs without significantly impacting retention in the upper airways.

In pathophysiological conditions, if there is impaired mucociliary advection, then it may lead to significantly reduced *Pe*_*d*_. Such a situation would promote drug retention in the upper airways since the time-scale for pure diffusive drug transport would be extremely long.

### Effect of breathing time period on drug deposition and retention

Deposition of drug-laden aerosols and drug retention in the lungs also depends on the breathing time period *T*_*b*_ through two parameters–the airway Strouhal number *St*_*a*_ (Eq 6) and the mucus Strouhal number *St*_*m*_ (Eq 9). *St*_*a*_ is the ratio of the advective time scale of airflow to the breathing time period (see Eq 5). A longer breathing time period leads to lower *St*_*a*_. Keeping all other parameters the same, long breaths are deeper and lead to greater volume being inhaled. Consequently, the fraction of drug-laden aerosols deposited in the deep lungs are observed to increase as *St*_*a*_ decreases (see Fig 3C). Correspondingly, *ϕ*_*d*_ increases and shifts towards deeper airways (see Fig 4B). It is seen that *ϕ*_*d*_ remains substantial in the deep lungs when *St*_*a*_ ≤ 0.01, but becomes negligible when *St*_*a*_ ≥ 0.05 (see *Fig D in* S1 Text for more details).

The breathing time period *T*_*b*_ also impacts the mucus Strouhal number (*St*_*m*_), which is the ratio of the mucociliary advection to breathing time scales (see Eq 8). A longer breathing time period relative to the time scale of mucociliary advection leads to lower *St*_*m*_, which implies greater advective clearance of the mucus in a breathing cycle. Thus, longer breaths inhibit drug retention (see Fig 4C). This is particularly evident from the drug washout curve at *N* = 0 (see Fig 4C inset). However, lower drug retention is observed to remain limited to the upper airways and does not influence drug retention in the deep lungs (see *Fig E in* S1 Text for more details).

In summary, on the one hand, longer breath time period leads to deep lungs deposition of drugs, which is good. On the other hand, it also inhibits drug retention in the upper airways, which is bad. These conflicting outcomes can be resolved by noting that longer breaths do not affect drug retention in the deep lungs. Achieving deep lung deposition is more critical. Shorter breathing times or shallow breaths can reduce deep lungs deposition of the drug-laden aerosols. Similar observations have also been made in experimental investigations carried out by Mallik et al. [25].

### Effect of exposure time

The impact of exposure duration (*τ*_*exp*_) is studied by varying the number of breathing cycles for which the lungs are assumed to be exposed to the drug-laden aerosols at the inlet of *N* = 0 generation. It is observed that the aerosol deposition pattern within the lungs remains almost identical with increase in *τ*_*exp*_, but the magnitude of aerosol deposition (*S*_*d*_) (and hence *ϕ*_*d*_) increases as *τ*_*exp*_ become longer (see *Fig F in* S1 Text). This increases the washout time causing longer retention of drugs in the lungs. It is found that the increase in *S*_*d*_ and *ϕ*_*d*_ with *τ*_*exp*_ is linear, as shown in Fig 4D. This information can be used to estimate the exposure time required for achieving a required drug concentration in various regions of the lungs or to estimate the drug dose delivered to a particular region of the lung over a specific exposure time (see S1 Text for more details).

### Drug delivery efficacy

Pulmonary drug delivery systems have a major drawback since majority of the inhaled aerosolized drugs get deposited in the mouth and the pharynx. Only about 5–12% of the inhaled drugs reach the trachea for further inhalation into the respiratory tract [26]. This often leads to prescription of larger drug doses in order to obtain the required health effects. Larger drug doses can, however, lead to side effects and the drug dose prescribed should, therefore, be minimized as much as possible. The present study helps in identifying plausible routes for enhancing the efficacy of drug delivery to the lungs and thereby, minimizing the inhaled drug dose.

For example, consider the delivery of salbutamol from a pressurised meter-dose inhalers (100 *μ*g per puff) in an asthmatic child. It is estimated using the present analysis that only 2.8 *μ*g (out of 100 *μ*g) per puff of aerosolised salbutamol i.e. 2.8% of the inhaled drugs reach the deep lung considering the size of the aerosolized drugs to be 3 *μ*m (corresponding deposition fraction of 28%) and 10% inhaled aerosols reaching the trachea (see Table 1). Aerosols generated from inhalers are in the range of 1–5 *μ*m. The corresponding salbutamol concentration in blood is estimated to be 42.26 ng/ml after 40 inhaler puffs assuming the total deposited drugs in the deep lung to remain available to blood circulation (see S1 Text for more details). 20–40 puffs, corresponding to 20–40 ng/ml of salbutamol in blood, are usually required to reverse the effects of bronchoconstriction in children [26]. The present analysis can, thus, be used to obtain a close estimate of the physiologically measured drug concentration. This can be used to gauge the efficacy of drug delivery for various combination of the pertinent parameters.

**Table 1:** Comparison of drug dose delivered to the deep lung for various aerosol sizes and breathing periods. The aerosols carry the drugs and are generated from inhalers. It is assumed that 10% of the aerosols inhaled reach the trachea for further inhalation into the deep lung [26]. Enhancement is calculated with respect to 3 *μ*m aerosols for 4s breathing period.

Inhaled Dose per puff (*μ*g)	Aerosol Size (*μ*)	Breathing Period (*s*)	Drug dose reaching deep lung per puff (*μ*g)	Enhancement (%)
100	0.02	4	3.95	41
	0.5		1.41	−49.6
	3		2.8	n/a
	10		0.36	−87.1
	3	2	0.52	−81.4
		4	2.8	n/a
		8	5.38	92.14
		16	6.39	128.21

The present analysis shows that a plausible way of increasing the efficacy of drug delivery to the deep lung is by controlling the size of the inhaled aerosols generated using inhalers/nebulizers. Drug delivery to the deep lung is observed to be reduced significantly if the corresponding aerosol size is larger than 5 *μ*m or smaller than 1 *μ*m (see Table 1). Aerosols larger than 5 *μ*m deposit mainly in the upper airways due to impaction, while those smaller than 1 *μ*m mostly remain suspended and are exhaled out resulting in lower deposition in the lung [27]. However, drug delivery to the deep lung increases substantially if 0.02 *μ*m aerosols are inhaled (see Table 1 and *Fig B in* S1 Text for more details) due to more efficient diffusional deposition of aerosols smaller than 0.1 *μ*m [27]. As such, drug delivery to the deep lung could be enhanced if such small aerosols are used. However, aerosols in this size range are impractical in the context of drug delivery systems because of the large energy requirement for generation of such aerosols [27].

Controlling the time period of breathing while taking inhaler puffs (or using nebulizers) is another strategy which can be adopted to increase deep lung drug deposition. The present analysis shows that for longer breaths (see Table 1) drug deposition increases significantly in the deep lung. Slow and deep breathing while inhaling the drugs can, as such, enhance the efficacy of deep lung drug deposition. This is the reason why it is recommended to breathe deeply and slowly while using inhalers/nebulizers [28].

## Summary

The present analysis uses a coupled aerosol (airway)-drug (mucus) flow model to study the deposition and retention of drug-laden aerosols in the lungs. While the computational model is simplified and makes several assumptions (as discussed in the sections on mathematical model and lung geometry), it is still a tractable model which can be used to capture the key trends of drug deposition and retention considering the entire lungs. This is the best that can be achieved given the overwhelming complexity of aerosol aerodynamics in the respiratory tract, when the whole lung is considered, across wide ranges of particle sizes, flow patterns, anatomical variability, and stochastic nature of the problem. The model provides useful quantitative results which can be utilised to identify plausible means and mechanisms for enhancing the efficacy of drug delivery to the lungs. The major observations and predictions obtained using this model are summarized below -

- The probability of deep lung (alveolar) deposition of inhaled aerosols is observed to be large for aerosols smaller than 10 *μ*m and larger than 0.003 *μ*m under normal breathing conditions. Within this range, deposition is observed to vary non-monotonically with maximum deposition occurring for 0.02 *μ*m aerosols. These observations are similar to that reported by various other researchers [4, 9, 10]. It is to be noted that the predicted aerosol size range for deep lung deposition is dependent on the breathing conditions.
- Deep lung deposition of aerosols is observed to become larger with increase in breathing period, similar to the experimental observations of Mallik et al. [25].
- A longer exposure is also observed to lead to larger amount of aerosol deposition in the lungs. Larger deposition requires a longer washout period and hence, drug retention in the lungs becomes enhanced with an increase in exposure.
- Retention of the deposited drug molecules in the upper (proximal) airways is observed to be dependent on the breathing period and the mucociliary clearance rate. Longer breath and a faster mucociliary clearance rate are observed to inhibit drug retention in the upper airways.
- In the deep (distal) lung, drug molecule retention is observed to be dependent only on diffusivity of the drug molecules in the mucus. Thus, smaller drug molecules and lower mucus viscosity (both of which increase diffusivity) inhibits drug retention in the deep lung by promoting quicker washout of the deposited drugs and vice-versa.
- Drug delivery efficacy in the deep lung is observed to be maximum for aerosols in the size range of 1–5 *μ*m. Focused investigations need to be carried out for improving the design of aerosol generators in order to obtain consistent aerosol production in the above size range. Although greater efficacy is obtained for very fine aerosols (<0.1 *μ*m), production of such aerosols is impractical in the context of pulmonary drug delivery.
- It is also observed that the amount of drugs deposited in the deep lung increases by a factor of 2 when the breathing time period is doubled, with respect to normal breathing, suggesting that breath control can be explored as a mechanism to increase drug delivery efficacy in the deep lung.

## Supporting information

S1 Text*Supplementary Material* for *Pulmonary drug delivery and retention: a computational study to identify plausible parameters based on a coupled airway-mucus flow model* with additional details on the lung geometry, detailed derivation and validation of the mathematical model and supporting results. **Table A**: *Parameters used in modelling the lung geometry*. **Table B**: *Fractions of alveolated airways in different generations*. **Fig A**: *Comparison of the diffusional deposition probability using the simplified model (B_d_, k_d_) used in the present study and the model proposed by Yeh & Schaum^10^ for a aerosol diameter of* 0.1 *μm*. **Fig B**: *Comparison of the calculated deposition fraction (DF) of inhaled aerosols for (a) the whole lungs and (b) the alveolar region with the experimental results obtained by Heyder et al.^11^ for different aerosol diameter (d_a_), and comparison of the impact of different deposition mechanisms as a function of aerosol diameter in (c) the whole lung and (d) the alveolar region*. **Fig C**: *(a) Aerosol deposition* ($S_{d}=L_{D}^{\prime }\phi_{a}$) *within the lungs for different Pe_a_ (b) Temporal change in drug concentration (ϕ_d_) at N* = 0 *for different Pe_a_ (c) Drug concentration within the lungs at τ* = 10000 *for different Pe_a_. The results are shown for St_a_* = 0.0095, *Pe_d_* = 4.56 × 10^7^, *St_m_* = 359.7122, *τ_exp_* = 5. **Fig D**: *(a-b) Aerosol deposition* ($S_{d}=L_{D}^{\prime }\phi_{a}$) *within the lungs for different St_a_ (c) Temporal change in drug concentration (ϕ_d_) at N* = 0 *for different St_a_ (d) Drug concentration (ϕ_d_) within the lung for different St_a_ at τ* = 10000. *The results are shown for Pe_a_* = 2.85 × 10^10^, *Pe_d_* = 4.56 × 10^7^, *St_m_* = 359.7122, *τ_exp_* = 5. **Fig E**: *Drug concentration (ϕ_v_) within the lungs for various St_m_ at (a) the end of aerosol exposure (τ* = 5) *and (b) at τ* = 10000. *The results are shown for Pe_a_* = 2.85 × 10^10^, *Pe_d_* = 4.56 × 10^7^, *St_a_* = 0.0095, *τ_exp_* = 5. **Fig F**: *(a) Total aerosol deposition* ($S_{d}=L_{D}^{\prime }\phi_{a}$) *within the lung for different τ_exp_. Deposition for τ_exp_* = 5–100 *is additionally shown as inset to ensure proper readability (b) Drug concentration (ϕ_d_) within the lungs for different τ_exp_ at the end of exposure i.e. at τ* = *τ_exp_. The results are shown for Pe_a_* = 2.85 × 10^10^, *Pe_d_* = 4.56 × 10^7^, *St_a_* = 0.0095, *St_m_* = 359.7122.(PDF)
