# Supplementary material for: Pulmonary drug delivery and retention: A computational study to identify plausible parameters based on a coupled airway-mucus flow model
Source: PLoS Comput Biol. 2022 Jun 2;18(6):e1010143. doi: 10.1371/journal.pcbi.1010143 (PMC9197018; doi:10.1371/journal.pcbi.1010143)
Supplement: S1 Text — Supplementary Material for Pulmonary drug delivery and retention: a computational study to identify plausible parameters based on a coupled airway-mucus flow model with additional details on the lung geometry, detailed derivation and validation of the mathematical model and supporting results. Table A: Parameters used in modelling the lung geometry. Table B: Fractions of alveolated airways in different generations. Fig A: Comparison of the diffusional deposition probability using the simplified model (Bd, kd) used in the present study and the model proposed by Yeh & Schaum10 for a aerosol diameter of 0.1 μm. Fig B: Comparison of the calculated deposition fraction (DF) of inhaled aerosols for (a) the whole lungs and (b) the alveolar region with the experimental results obtained by Heyder et al.11 for different aerosol diameter (da), and comparison of the impact of different deposition mechanisms as a function of aerosol diameter in (c) the whole lung and (d) the alveolar region. Fig C: (a) Aerosol deposition (Sd=LD′ϕa) within the lungs for different Pea (b) Temporal change in drug concentration (ϕd) at N = 0 for different Pea (c) Drug concentration within the lungs at τ = 10000 for different Pea. The results are shown for Sta = 0.0095, Ped = 4.56 × 10^7^, Stm = 359.7122, τexp = 5. Fig D: (a-b) Aerosol deposition (Sd=LD′ϕa) within the lungs for different Sta (c) Temporal change in drug concentration (ϕd) at N = 0 for different Sta (d) Drug concentration (ϕd) within the lung for different Sta at τ = 10000. The results are shown for Pea = 2.85 × 10^10^, Ped = 4.56 × 10^7^, Stm = 359.7122, τexp = 5. Fig E: Drug concentration (ϕv) within the lungs for various Stm at (a) the end of aerosol exposure (τ = 5) and (b) at τ = 10000. The results are shown for Pea = 2.85 × 10^10^, Ped = 4.56 × 10^7^, Sta = 0.0095, τexp = 5. Fig F: (a) Total aerosol deposition (Sd=LD′ϕa) within the lung for different τexp. Deposition for τexp = 5–100 is additionally shown as inset to ensure proper readabili [file pcbi.1010143.s001.pdf]

S1 Text

Supplementary Materials

for

Pulmonary drug delivery and retention: a computational study to identify plausible parameters based on a coupled airway-mucus flow model

Aranyak Chakravarty,[1,2] Mahesh V. Panchagnula,[2] Alladi Mohan,[3] and Neelesh A. Patankar[4,a)]

[1)] *School of Nuclear Studies & Application, Jadavpur University, Kolkata, India*

[2)] *Department of Applied Mechanics, Indian Institute of Technology Madras, Chennai, India*

[3)] *Department of Medicine, Sri Venkateswara Institute of Medical Sciences, Tirupati, India*

[4)] *Department of Mechanical Engineering, Northwestern University, Evanston, Illinois, United States of America*

[a)] Electronic mail: n-patankar@northwestern.edu

I. IDEALIZATION OF THE LUNG GEOMETRY

Table A summarises the magnitudes of various parameters used while approximating the lung geometry (*see Eqs 1-3 in the main manuscript)*. Table B lists the assumed fraction of airway area (γ) that is alveolated at each generation in the lung model. This is required in calculating aerosol deposition in the alveolar region (see Eqs S32 and S34).

TABLE A. Parameters used in modelling the lung geometry

L_0	0.12 m[1]	α	0.73
A_0	0.000317m^2[1]	β	0.71
R_0	$\sqrt{A_0/\pi}$	ζ	0.9
δ_0	10 μm[2]	ε	0.87
$A_{m,0}$	$2\pi R_0 \delta_0$		
$V_{m,0}$	-5 mm/min[2]		

TABLE B. Fractions of alveolated airways in different generations[3]

Lung Generation (N)	Fraction of alveolated area (γ)
0-16	0
17	0.0011
18	0.0041
19	0.0135
20	0.0509
21	0.1168
22	0.2712
23	0.5424

II. MATHEMATICAL MODEL

A. Aerosol transport

The one-dimensional transport equation for aerosols at any location in the idealised lung geometry is expressed as

$$\frac{\partial(Ac_a)}{\partial t} + \frac{\partial(Qc_a)}{\partial x} = \frac{\partial}{\partial x}\Big(AD_a\frac{\partial c_a}{\partial x}\Big) - L_D c_a, \tag{S1}$$

where, c_a represents the aerosol concentration, Q represents the volume flow rate of air in breathing, and D_a represents the diffusivity of aerosols in air. Eq S1 is equivalent to Eq 4 in the main manuscript. The coefficient L_D accounts for the aerosols deposited in the airway mucus. This equation is based on the *trumpet* model proposed by Taulbee & Yu[4] which has been later used by various authors to study different aspects of aerosol deposition in the lung[5–7]. The transport equation is formulated based on the assumption that the aerosols are monodispersed, do not undergo coagulation, and are decoupled from airflow in the lungs. It is also assumed that external forces (such as electrical and magnetic forces) do not have any influence on the aerosol dynamics. It is further assumed that there is no additional source of aerosols present within the lungs and the aerosols are either deposited in the airway mucus or washed out of the airways.

Eq S1 is presented in terms of airway length (x), while the lung model adopted (see *Fig 1 in the main manuscript*) is in terms of lung generation number (N). As such, Eq S1 needs to be converted to a more appropriate form in terms of N. This requires an additional mathematical relation (Eq S2) connecting airway length x and the lung generation number (N) given by

$$H = \frac{\partial N}{\partial x} = -\frac{1-\alpha}{L_0\alpha\,\ln(\alpha)\alpha^N}. \tag{S2}$$

which can be derived by considering Eq 2 in the main manuscript. Converting Eq S1 using Eqs S2 and $A_N = A_0(2\beta)^N$, we get

$$A_0(2\beta)^N\frac{\partial c_a}{\partial t} = H\frac{\partial}{\partial N}\Bigg[\Big(A_0(2\beta)^N D_a H\frac{\partial c_a}{\partial N}\Big) - \Big(Q_{max}q(t)c_a\Big)\Bigg] - L_D c_a, \tag{S3}$$

where, $q(t)$ represents the temporal sinusoidal function accounting for airflow variation during breathing such that $Q = Q_{max}q(t)$. Eq S3 is reduced to its dimensionless form by

multiplying and dividing Eq S3 with $\left(\dfrac{L_0}{A_0 D_a}\right)$ and $\left(-\dfrac{\alpha\,\ln(\alpha)}{1-\alpha}\right)$, respectively, and using the following scaling parameters

$$\tau = \frac{t}{T_b}, \phi_a = \frac{c_a}{c_{a,0}}, T_a = \frac{L_0 A_0}{|Q_{max}|}, St_a = \frac{T_a}{T_b}, Pe_a = \frac{|Q_{max}| L_0}{A_0 D_a}, D_a = \frac{k_B T C_S}{3\pi \mu_a d_a}, \tag{S4}$$

where, Pe_a and St_a are the Peclet number for aerosols and Strouhal number for the airways, respectively. ϕ_a and τ denotes the dimensionless aerosol concentration and time, respectively, while the quantities T_a and T_b represents the convective airflow time-scale and breathing time-scale, respectively. The expression of D_a is based on the Stokes-Einstein relation[8], where C_s represents the Cunningham slip correction, T represents the ambient temperature, μ_a denotes air viscosity, and d_a denotes the aerosol diameter.

The dimensionless equation (equivalent to Eq 6 in the main manuscript), thus, obtained is used to analyse aerosol transport in the present study and is given by

$$|Pe_a| St_a (2\alpha\beta)^N \frac{\partial(\phi_a)}{\partial \tau} = \frac{\partial F_a}{\partial N} - L'_D \phi_a, \tag{S5}$$

where, L'_D represents the dimensionless form of aerosol deposition coefficient (L_D; see Section II B) and F_a represents the total aerosol flux. These are expressed as follows -

$$L'_D = L_D \frac{L_0^2}{A_0 D_a} \alpha^N \tag{S6}$$

$$F_a = \left[\left(\left(\frac{2\beta}{\alpha}\right)^N \left(\frac{1-\alpha}{\alpha(\ln\alpha)}\right)^2 \frac{\partial \phi_a}{\partial N}\right) + \left(|Pe_a| q(t) \Big(\frac{1-\alpha}{\alpha\,\ln(\alpha)}\Big) \phi_a\right)\right]. \tag{S7}$$

B. Aerosol deposition models

The major mechanisms of aerosol deposition in the lungs have been identified in the literature as diffusion, sedimentation and impaction of the aerosols in the airways, as well as diffusion and sedimentation of the aerosols in the alveoli[5,9]. Different empirical models have been used in the past to estimate various depositions. However, these models need to be converted into a form consistent for use with the derived transport equation (Eq S5). This is discussed in the present section.

The probability of aerosol deposition in the airways by diffusion (P_d), sedimentation (P_s) and impaction (P_i) can be expressed following Yeh & Schaum[10] as

$$\frac{c_{a,0} - c_a}{c_{a,0}} = P_d + P_s + P_i - P_dP_s - P_dP_i - P_sP_i - P_dP_sP_i \tag{S8}$$

Eq S8 can be re-written as

$$\begin{aligned} \frac{c_a}{c_{a,0}} &= (1-P_d)(1-P_s)(1-P_i) = (B_d e^{-k_d x})(B_s e^{-k_s x})(B_i e^{-k_i x}) \\ \implies c_a &= c_{a,0}\left[B_d B_s B_i e^{-(k_d+k_s+k_i)x})\right] \end{aligned} \tag{S9}$$

where, the terms B_d, B_s and B_i are the corresponding coefficients, and k_d, k_s and k_i are the corresponding constants in the exponential functions for different deposition mechanisms as proposed by Yeh & Schaum[10]. Detailed expressions for the different deposition mechanisms can be found in the subsequent discussion. Taking the derivative of c_a (Eq S9) with respect to x, we obtain -

$$\frac{dc_a}{dx} = -c_{a,0}\left[(k_d + k_s + k_i)B_d B_s B_i e^{-(k_{d,n}+k_s+k_i)x})\right] = -(k_d + k_s + k_i)c_a \tag{S10}$$

Equation S10 represents the droplet deposition flux in the airways. It is further converted to a dimensionally relevant form for use in the transport equation (Eq S3) as follows -

$$\begin{aligned} \frac{D(Ac_a)}{Dt} &\simeq Av\frac{dc_a}{dx} = -Av(k_d + k_s + k_i)c_a = L_D c_a \\ &\implies L_D = Av(k_d + k_s + k_i) \end{aligned} \tag{S11}$$

The term L_D represents the aerosol deposition coefficient which is determined using different empirical relations. The empirical relations are converted to a form relevant to Eq S11 and then reduced to their dimensionless forms for use in the final transport equation (Eq S5). These are discussed in the following sections for the various deposition mechanisms considered in this analysis.

1. Diffusional deposition in the airways

The probability of diffusional deposition (P_d) of the aerosols in the airways can be expressed following Yeh & Schaum[10] as

$$P_d = 1 - 0.819e^{-7.315Gx} - 0.0976e^{-44.61Gx} - 0.0325e^{-114Gx}$$
$$= 1 - B_{d,1}e^{-k_{d,1}x} - B_{d,2}e^{-k_{d,2}x} - B_{d,3}e^{-k_{d,3}x} \quad \text{(S12)}$$

where, $G = \dfrac{D_a}{2R_N^2 v_N}$. The above equation can be simplified by expressing the coefficients in terms of effective magnitudes (B_d, k_d) as follows

$$P_d = 1 - B_d e^{-k_d x} \quad \text{(S13)}$$

where, B_d and k_d are determined as

$$B_d = B_{d,1} + B_{d,2} + B_{d,3}$$
$$k_d = k_{d,1} + k_{d,2} + k_{d,3} \quad \text{(S14)}$$

It is estimated that this simplification does not have any significant influence on the calculation for diffusional deposition (see Fig A). The simplified form is, as such, used in this analysis for calculation diffusional deposition in the airways.

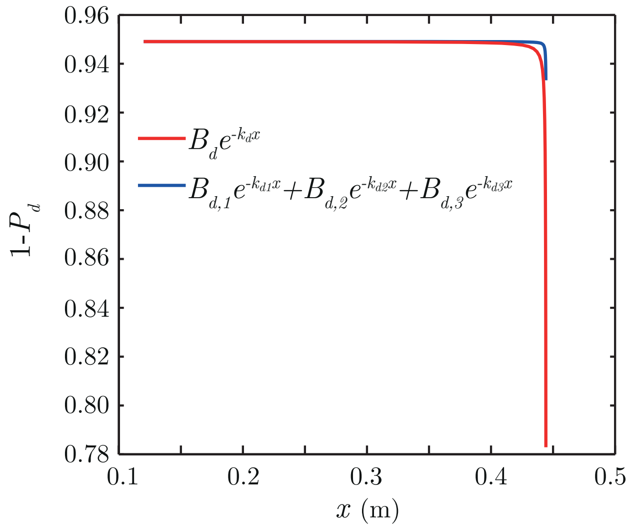

FIG. A. Comparison of the diffusional deposition probability using the simplified model (B_d, k_d) used in the present study and the model proposed by Yeh & Schaum[10] for a aerosol diameter of 0.1 μm.

Using Eqs S12 and S13, we obtain -

$$k_{d,1} = 7.315\frac{D_a}{2R_N^2 v_N}, k_{d,2} = 44.61\frac{D_a}{2R_N^2 v_N}, k_{d,3} = 114\frac{D_a}{2R_N^2 v_N} \quad \text{(S15)}$$

and

$$k_d = k_{d,1} + k_{d,2} + k_{d,3} = (7.315 + 44.61 + 114)\frac{D_a}{2R_N^2 v_N} \tag{S16}$$

where, R_N and v_N denotes the airway radius and airflow velocity of a particular lung generation, respectively. D_a denotes aerosol diffusivity in air. Using this, droplet deposition in the airways due to aerosol diffusion is estimated as (refer to Eq S11)

$$L_{D,d} = v_N A_{N,T} k_d = v_N \pi R_N^2 2^N (7.315 + 44.61 + 114)\frac{D_a}{2R_N^2 v_N} \tag{S17}$$

Conversion of Eq S17 to its dimensionless form gives us the following expression for dimensionless diffusional deposition of the aerosols in the airways -

$$L'_{D,d} = L_{D,d}\frac{L_0^2}{A_0 D_a}\alpha^N = \left(\frac{L_0}{R_0}\right)^2 (2\alpha)^N (3.66 + 22.305 + 57) \tag{S18}$$

2. Sedimentation deposition in the airways

The probability of deposition of the aerosols (P_s) due to sedimentation in the airways is expressed following Yeh & Schaum[10] as

$$P_s = 1 - exp\left[-\left(\frac{gC_s\rho_a d_a^2 \cos(\psi_N)}{9\pi\mu_{air} R_N v_N} x\right)\right] \tag{S19}$$

where, ρ_a, g and ψ represents droplet density, gravitational acceleration and airway orientation angle considering horizontal as 90°, respectively. Linearising the above equation using the approach followed in Eq.S9, we obtain -

$$k_s = \frac{gC_s\rho_a d_a^2 \cos(\psi_N)}{9\pi\mu_{air} R_N v_N} \tag{S20}$$

Aerosol deposition in the airways due to sedimentation can, then, be estimated as -

$$\begin{aligned} L_{D,s} = v_N A_{N,T} k_s &= v_N(\pi R_N^2 2^N)\frac{gC_s\rho_a d_a^2 \cos(\psi_N)}{9\pi\mu_{air} R_N v_N} \\ &= \frac{1}{9}\frac{R_N gC_s\rho_a d_a^2 \cos(\psi_N)}{\pi\mu_{air}} 2^N \end{aligned} \tag{S21}$$

Conversion of the dimensional deposition ($L_{D,s}$) to its dimensionless form gives us the following expression for dimensionless sedimentation deposition in the airways -

$$L'_{D,s} = L_{D,s}\frac{L_0^2}{A_0 D_a}\alpha^N = \frac{1}{3}\left(\frac{L_0}{R_0}\right)^2 \left(2\alpha\sqrt{\beta}\right)^N S_g \cos(\psi_N) \tag{S22}$$

where, S_g is defined as the sedimentation parameter and expressed as

$$S_g = \frac{R_0 \rho_a d_a^3 g}{k_B T} \tag{S23}$$

3. Impact deposition in the airways

The probability of deposition due to impaction (P_i) of the aerosols in the airways is given by Yeh & Schaum[10] as

$$P_i = 1 - f_i(\theta, St) \tag{S24}$$

where, θ denotes the branching angle of the airways and St denotes the Stokes number $(= \frac{C_s \rho_a r_a^2 v_N}{9\mu_{air} R_N})$. The function $f_i(\theta, St)$ is expressed as follows -

$$\begin{aligned} f_i(\theta, St) &= \frac{2}{\pi}\cos^{-1}(\theta \cdot St) - \frac{1}{\pi}\sin\Big[2\cos^{-1}(\theta \cdot St)\Big] \text{ for } \theta \cdot St < 1 \text{ (Inhalation)} \\ &= 1 \text{ for } \theta \cdot St \geq 1 \text{ (Exhalation)} \end{aligned} \tag{S25}$$

The expression of P_i is not in a form that can be directly linearized. As such, certain mathematical treatments are carried out in order to estimate the impact deposition. Loss of aerosols i.e. the amount of aerosols deposited in one generation of the lungs can be determined based on the aerosol concentrations before and after the lung generation. Mathematically, this can be expressed as

$$\begin{aligned} \text{Loss in a generation} &= \frac{c_{bef} - c_{aft}}{c_{bef}} = 1 - f_i(\theta, St) \\ \implies \frac{c_{aft}}{c_{bef}} &= f_i(\theta, St) \end{aligned} \tag{S26}$$

In terms of lung generations, the above expression can be re-written as

$$c_d = f_i^N(\theta, St) c_{a,0} \tag{S27}$$

Differentiating with respect to generation number, we obtain -

$$\frac{dc_a}{dN} = c_{a,0}\ln(f_i^N(\theta, St)) f_i^N(\theta, St) = \ln(f_i^N(\theta, St)) c_a \tag{S28}$$

Converting the above derivative to a derivative in terms of x, we get -

$$\begin{aligned} \frac{dc_a}{dx} &= -(-\ln(f_i^N(\theta, St)))\frac{dN}{dx} c_a \\ \implies k_i &= (-\ln(f_i^N(\theta, St)))\frac{dN}{dx} \end{aligned} \tag{S29}$$

The impact deposition is estimated using the above expression as

$$L_{D,i} = v_N A_{N,T} k_i = v_N \pi R_N^2 2^N (-\ln(f_i^N(\theta, St))) \frac{dN}{dx} \tag{S30}$$

The dimensionless form of the impact deposition is obtained following a similar approach as in Sections II B 1 and II B 2. The dimensionless impact deposition, thus, obtained is expressed as

$$L'_{D,i} = L_{D,i} \frac{L_0^2}{A_0 D_a} \alpha^N = |Pe_a| q(t) \ln(f_i^N(\theta, St)) \frac{(1-\alpha)}{\alpha \ln(\alpha)} \tag{S31}$$

4. Diffusional deposition in the alveoli

Diffusional deposition of the aerosols in the alveoli is estimated using the following dimensionless expression -

$$L'_{D,d,alv} = \gamma_N \eta_{d,alv} |Pe_a| q(t) \Big(\frac{1-\alpha}{-\alpha \ln(\alpha)} \Big) \tag{S32}$$

where, γ_N denotes the fraction of alveolated area in the corresponding generation (see Table B) and $\eta_{d,alv}$ denotes the diffusional deposition efficiency in the alveoli. $\eta_{d,alv}$ is expressed as[5]

$$\eta_{d,alv} = 1 - \frac{6}{\pi^2} \sum \frac{1}{k^2} exp\Bigg[- \frac{4k^2 t D_a}{d_{eq}^2} \Bigg] \tag{S33}$$

5. Sedimentation deposition in the alveoli

Deposition of the inhaled aerosols due to their sedimentation in the alveoli are estimated using the following dimensionless expression -

$$L'_{D,s,alv} = \gamma_N \eta_{s,alv} |Pe_a| q(t) \Big(\frac{1-\alpha}{-\alpha \ln(\alpha)} \Big) \tag{S34}$$

where, γ_N and $\eta_{s,alv}$ denotes the fraction of alveolated area in the corresponding generation (see Table B) and sedimentation deposition efficiency in the alveoli, respectively. $\eta_{s,alv}$ is expressed as[5]

$$\eta_{s,alv} = \Bigg[1 + \min\Big(\frac{d_s}{d_{eq}}, 1\Big) \Bigg]^2 \Bigg[1 - 0.5\min\Big(\frac{d_s}{d_{eq}}, 1\Big) \Bigg]^2 - 1 \tag{S35}$$

6. *Total deposition*

The total amount of aerosols deposited at any lung generation is determined as the sum total of aerosol deposition through all deposition mechanisms. Mathematically, this is expressed as

$$L'_D = L'_{D,d} + L'_{D,s} + L'_{D,i} + L'_{D,d,alv} + L'_{D,s,alv} \tag{S36}$$

C. Drug molecule transport in mucus

The corresponding 1D transport equation for the drugs deposited in the airway mucus (equivalent to Eq 7 in the main manuscript) is expressed as

$$\frac{\partial(A_m c_d)}{\partial t} + \frac{\partial(Q_m c_d)}{\partial x} = \frac{\partial}{\partial x}(A_m D_d \frac{\partial c_d}{\partial x}) + \text{Source} \tag{S37}$$

where, c_d denotes the drug concentration in the airway mucus, Q_m represents the volume flow rate of mucociliary clearance and D_d denotes the diffusivity of drug molecules in the mucus layer.

The drug-laden aerosols deposited in the airway mucus serve as the only source of drugs in the lungs. The source term in Eq S37 is, therefore, equivalent in magnitude to the deposition term in Eq S1 ($L_D c_a$) times the drug load in aerosols (ϕ_l). Mathematically, this is expressed as -

$$\text{Source} = L_D c_a \phi_l \tag{S38}$$

where, ϕ_l is defined as the amount of drug molecules contained by the aerosols per unit amount of the aerosols. Equation S37 is converted to a form in terms of N using Eqs S2 and $A_m = A_{m,0}(2\sqrt{\beta}\zeta)^N$ in a similar manner as in Section II A as follows -

$$A_{m,0}(2\zeta\sqrt{\beta})^N \frac{\partial c_d}{\partial t} = H \frac{\partial}{\partial N}\left[\left(A_{m,0}(2\zeta\sqrt{\beta})^N D_d H \frac{\partial c_d}{\partial N}\right) - \left(Q_{m,0}(2\varepsilon\zeta\sqrt{\beta})^N c_d\right)\right] + (\phi_l L_D c_a) \tag{S39}$$

The above equation is further reduced by multiplying and dividing by $\left(\frac{L_0}{A_{m,0}D_d}\right)$ and $\left(-\frac{\alpha\,\ln(\alpha)}{1-\alpha}\right)$, respectively. The reduced equation is expressed as

$$\frac{L_0|V_{m,0}|}{D_d}(2\alpha\zeta\sqrt{\beta})^N\frac{T_m}{T_b}\frac{\partial c_d}{\partial t} = \frac{\partial}{\partial N}\left[\left(\Big(\frac{2\zeta\sqrt{\beta}}{\alpha}\Big)^N\Big(\frac{1-\alpha}{\alpha\,\ln(\alpha)}\Big)^2\frac{\partial c_d}{\partial N}\right) - \left(\frac{L_0|V_{m,0}|}{D_d}(2\varepsilon\zeta\sqrt{\beta})^N c_d\right)\right] + \left(\phi_l L'_D\frac{A_0 D_a}{L_0^2\alpha^N}\phi_a c_{a,0}\frac{L_0^2\alpha^N}{A_{m,0}D_d}\right) \tag{S40}$$

The following parameters are utilised to achieve the dimensionless form of the drug molecule transport equation in the airway mucus given by Eq S42.

$$\tau = \frac{t}{T_b}, \phi_d = \frac{c_d}{c_{d,0}}, c_{d,0} = \phi_l c_{a,0}\frac{A_0}{A_{m,0}}, T_m = \frac{L_0}{|V_{m,0}|}, St_m = \frac{T_m}{T_b}, Pe_d = \frac{|V_{m,0}|L_0}{D_d}, D_d = \frac{k_B T}{3\pi\mu_m d_d} \tag{S41}$$

$$|Pe_d|(2\alpha\zeta\sqrt{\beta})^N St_m\frac{\partial\phi_d}{\partial\tau} = \frac{\partial F_d}{\partial N} + \Big(L'_D\frac{D_a}{D_d}\phi_a\Big) \tag{S42}$$

where, ϕ_d, Pe_d and St_m represents the dimensionless drug concentration, Peclet number for the drug molecules and Strouhal number for the mucus layer, respectively. T_m denotes the time-scale for mucociliary transport. Eq S42 is equivalent to Eq 9 in the main manuscript. Drug diffusivity (D_d) is estimated using the Stokes-Einstein relation where μ_m represents mucus viscosity and r_d represents size of the drug molecules. The term F_d in Eq S42 represents the total flux of the drug molecules and is expressed as

$$F_d = \left[\left(\Big(\frac{2\zeta\sqrt{\beta}}{\alpha}\Big)^N\Big(\frac{1-\alpha}{\alpha\,\ln(\alpha)}\Big)^2\frac{\partial\phi_d}{\partial N}\right) - \left(|Pe_d|(2\varepsilon\zeta\sqrt{\beta})^N\phi_d)\right)\right] \tag{S43}$$

D. Implementation of the model and validation

The mathematical model discussed in Sections II A-II C is implemented for computational analysis using MATLAB®. The governing transport equations(Eq S5 and Eq S42) are discretised following the finite-difference technique with a first-order upwind and central-difference scheme used for the advective and diffusive terms, respectively. The temporal terms are discretised using explicit forward differencing.

The implemented mathematical model is validated with respect to aerosol deposition within the lungs. Aerosol depositions predicted using the computational model are compared with the experimental data of Heyder et al.[11] with respect to deposition in the whole lungs

as well as deposition specifically in the alveolar region of the lungs. The results are shown in Fig Ba and Fig Bb. It can be observed that the computed aerosol deposition is in quite good agreement with the experimentally determined data. Fig Bc and Fig Bd represent the contribution of different deposition mechanisms considered in the present analysis in the whole lung as well as the alveolar region. The dominance of the different deposition mechanisms are similar to that observed from literature[12].

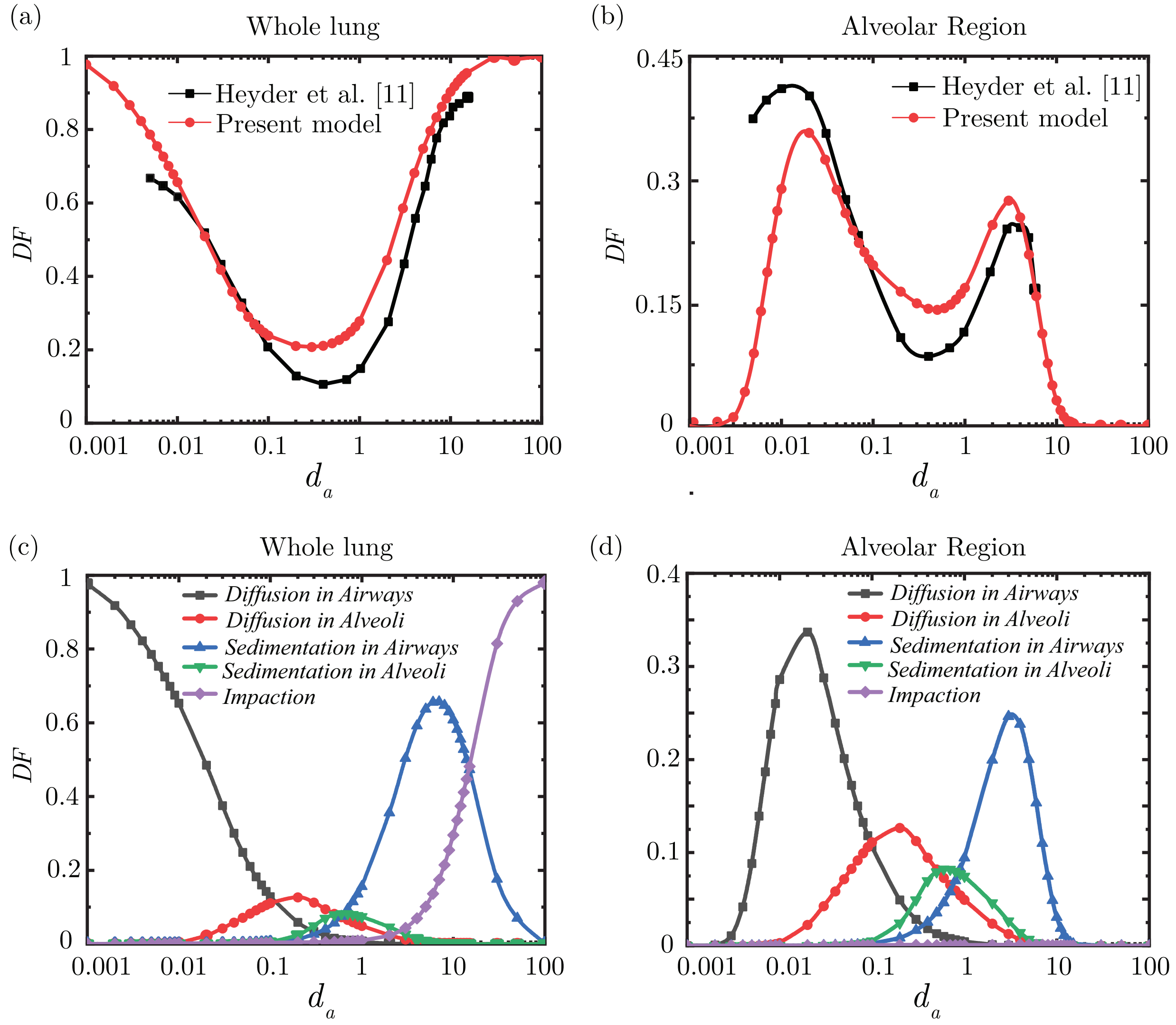

FIG. B. Comparison of the calculated deposition fraction (DF) of inhaled aerosols for (a) the whole lungs and (b) the alveolar region with the experimental results obtained by Heyder et al.[11] for different aerosol diameter (d_a), and comparison of the impact of different deposition mechanisms as a function of aerosol diameter in (c) the whole lung and (d) the alveolar region.

III. PHYSIOLOGICAL BASIS FOR PARAMETER SELECTION

The magnitudes of different parameters used in the mathematical model are selected based on relevant physiological data. Physiological quantities pertinent to the lung model are tabulated in Table A. Other relevant physiological quantities are summarised in Table C below.

TABLE C. Magnitudes of relevant physiological quantities considered in the study

Quantity	Magnitude	Quantity	Magnitude
d_a	0.01-20 μm	d_d	0.01-0.1 μm
μ_{air}	0.000018 kg/ms	μ_m	0.1 kg/ms
T	300 K	T_b	4 s
Q_{max}	0.0007925 m^3/s		

IV. SUPPORTING RESULTS

A. Effect of aerosol size on drug deposition in the deep lungs

Fig Ca shows the variation in aerosol deposition (S_d) within the lung with change in Pe_a. A larger volume of aerosols are able to reach the deeper generations of the lung with increase in Pe_a leading to larger aerosol deposition. This reverses when Pe_a is increased beyond 1.59×10^9 and at $Pe_a = 3.07\times10^{11}$, most of the aerosols are observed to get deposited in the first few generations and almost no deposition in the deep lung (beyond $N = 18$). Similar observations have been made in previous investigations as well[13]. The reason is due to a larger contribution of impact deposition of the aerosols in the earlier generations at such high Pe_a. The corresponding drug concentrations (ϕ_d) are shown in *Fig 3A* and *Fig 3B* in the main manuscript.

Fig Cb shows the temporal variation in ϕ_d at $N = 0$ of the lung. It can be observed that washout of the drugs from the lung does not undergo any significant temporal change with variation in Pe_a. However, the initial location of drug deposition within the lung is observed to have an important impact on its washout. Drugs deposited before $N = 18$ gets washed out quickly due to the stronger muco-ciliary clearance. Drugs deposited beyond $N = 18$,

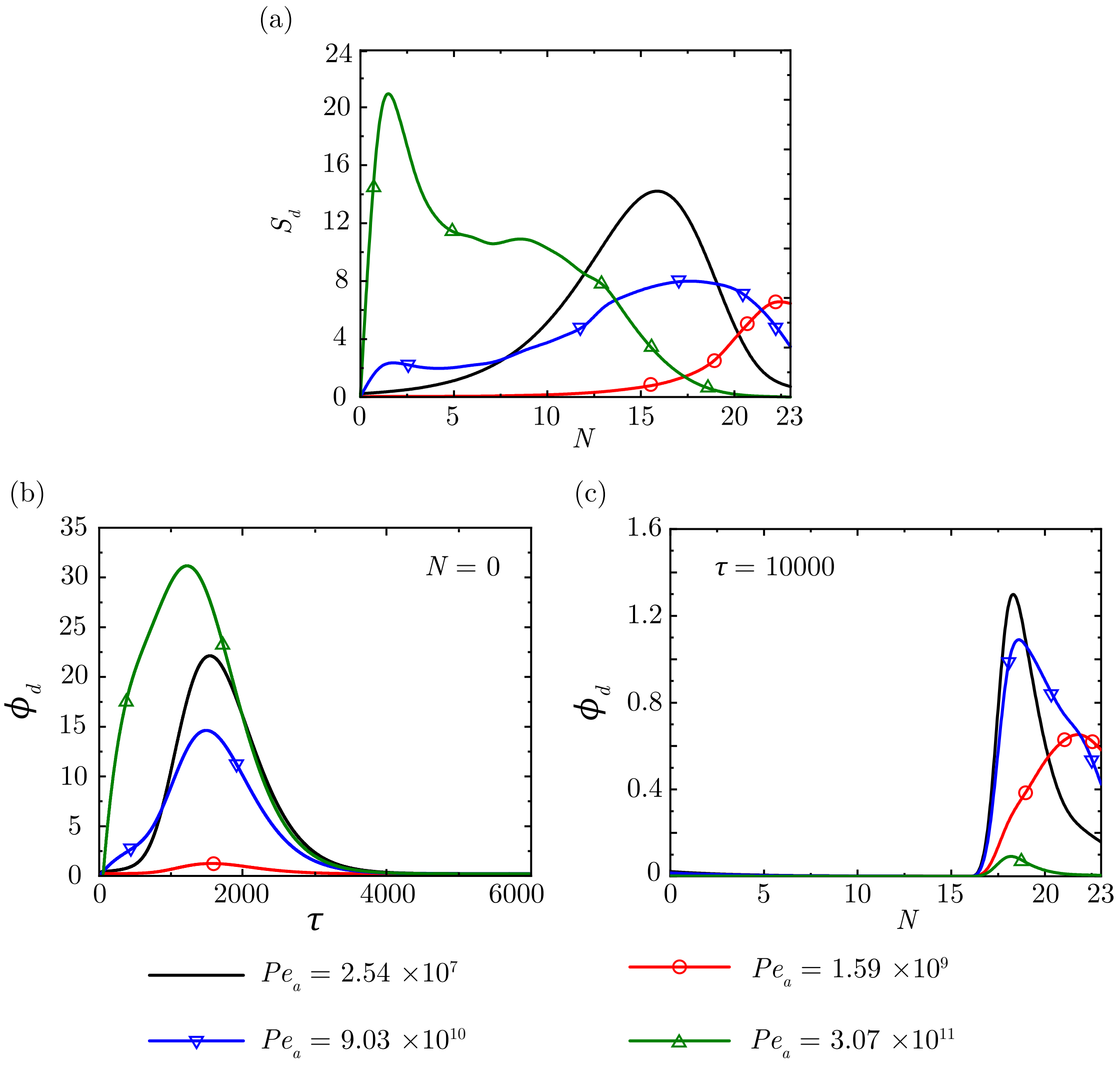

FIG. C. (a) Aerosol deposition ($S_d = L'_D \phi_a$) within the lungs for different Pe_a (b) Temporal change in drug concentration (ϕ_d) at $N = 0$ for different Pe_a (c) Drug concentration within the lungs at $\tau = 10000$ for different Pe_a. The results are shown for $St_a = 0.0095$, $Pe_d = 4.56 \times 10^7$, $St_m = 359.7122$, $\tau_{exp} = 5$.

however, gets transported much slowly due to the weak diffusive transport of the drug molecules in mucus in that region. Drugs deposited at very large Pe_a ($\sim 3.07 \times 10^{11}$) are, therefore, washed out of the lungs relatively quickly since majority of the deposition takes place before $N = 18$. At lower Pe_a, however, a substantial amount of the deposited drugs continue to persist in the deep lung (beyond $N = 18$) even though muco-ciliary clearance washes out the drugs from the upper generations. The retention of drugs in the deep lung is evident from the distribution of ϕ_d within the lung at $\tau = 10000$ in Fig Cc.

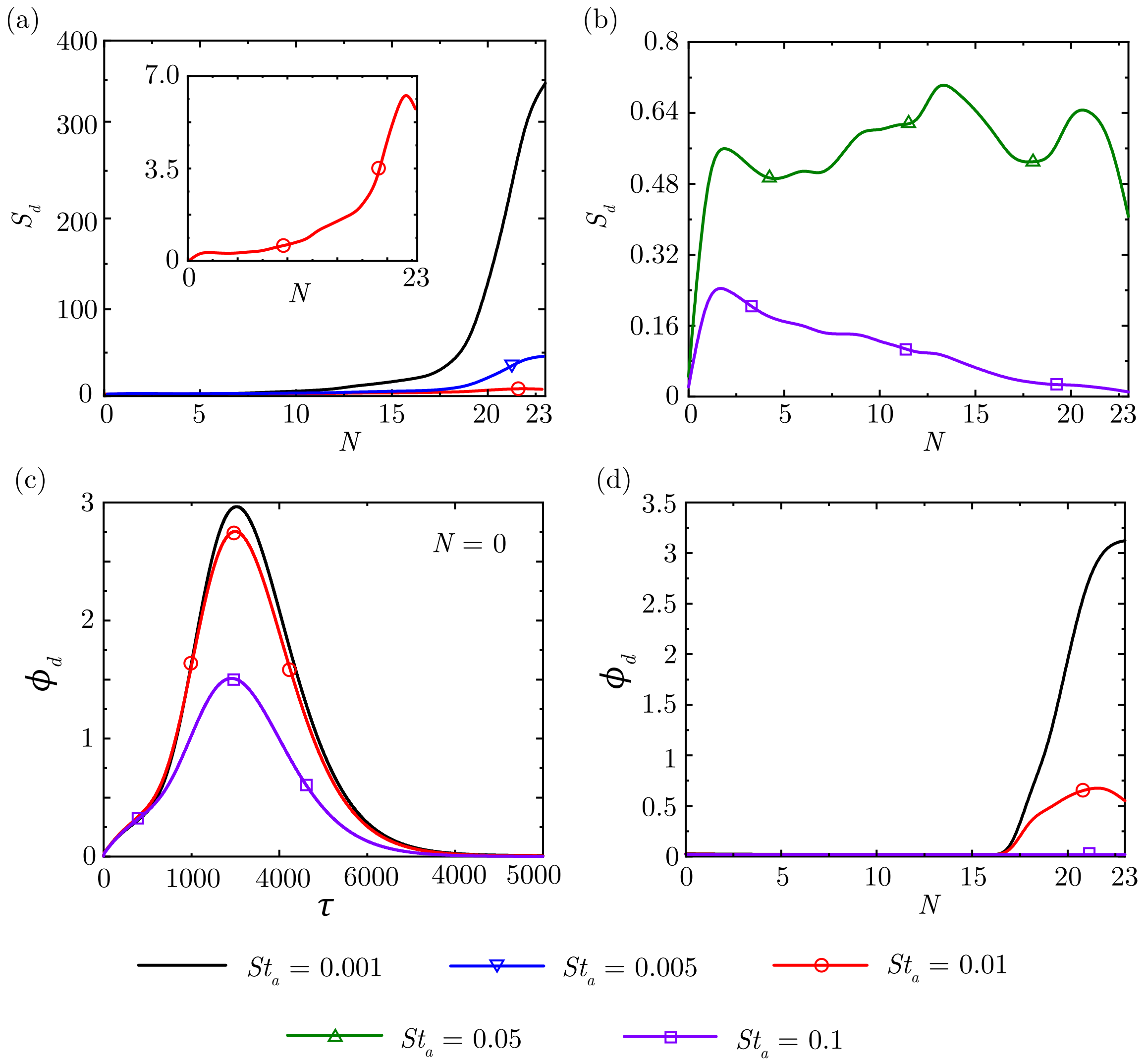

FIG. D. (a-b) Aerosol deposition ($S_d = L'_D \phi_a$) within the lungs for different St_a (c) Temporal change in drug concentration (ϕ_d) at $N = 0$ for different St_a (d) Drug concentration (ϕ_d) within the lung for different St_a at $\tau = 10000$. The results are shown for $Pe_a = 2.85 \times 10^{10}$, $Pe_d = 4.56 \times 10^7$, $St_m = 359.7122$, $\tau_{exp} = 5$.

B. Effect of breathing time period on drug deposition and retention

Fig Da and Fig Db highlights the change in aerosol deposition within the lung with variation in St_a. It can be observed that the magnitude of aerosol deposition in the mucus decreases and the deposition also tends to shift towards the upper airways with increase in St_a. This happens since the amount of aerosols being inhaled reduces with increase in St_a. The progression of ϕ_a front into the lung, therefore, decreases which, in turn, results

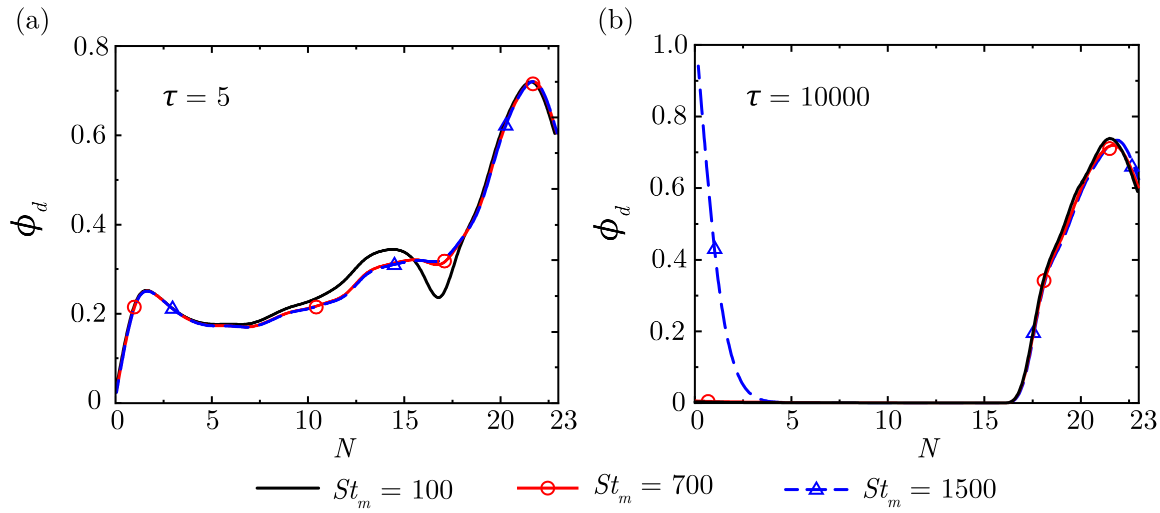

FIG. E. Drug concentration (ϕ_v) within the lungs for various St_m at (a) the end of aerosol exposure ($\tau = 5$) and (b) at $\tau = 10000$. The results are shown for $Pe_a = 2.85 \times 10^{10}$, $Pe_d = 4.56 \times 10^7$, $St_a = 0.0095$, $\tau_{exp} = 5$.

in the aforementioned change in aerosol deposition pattern. The corresponding change in drug concentration (ϕ_d) is shown in *Fig 4A* in the main manuscript.

Any change in St_a, however, do not affect the mucuociliary transport in the lung or drug diffusivity in the mucus. Drug washout from the lung, therefore, remains unaffected when St_a is changing, as shown from the temporal change of drug concentration at $N = 0$ in Fig Dc. Persistence of drugs in the deep lung is, as such, observed for the situations where deep lung deposition of drugs occur i.e. $St_a \leq 0.01$, as shown in Fig Dd.

Fig Ea represents ϕ_d at the end of aerosol exposure for various St_m. It can be observed that there is no significant difference between ϕ_d when St_m remains large. It is only when St_m becomes ~ 100 that deviations become apparent enough. The reason for these deviations is the much faster mucus clearance at low St_m which is able to transport the deposited drugs away from the initial deposition location even before the deposition is complete.

Fig Eb shows ϕ_d at $\tau = 10000$ for various St_m. It can be observed that there is a considerable difference between ϕ_d in the upper airways as a result of the varying rate of mucociliary transport. However, it is not observed to influence washout of the drugs from the deep lung in any manner. Breathing and muco-ciliary transport are, hence, observed to have no significant influence on drug washout from the deep lung.

C. Effect of exposure time on drug deposition and retention

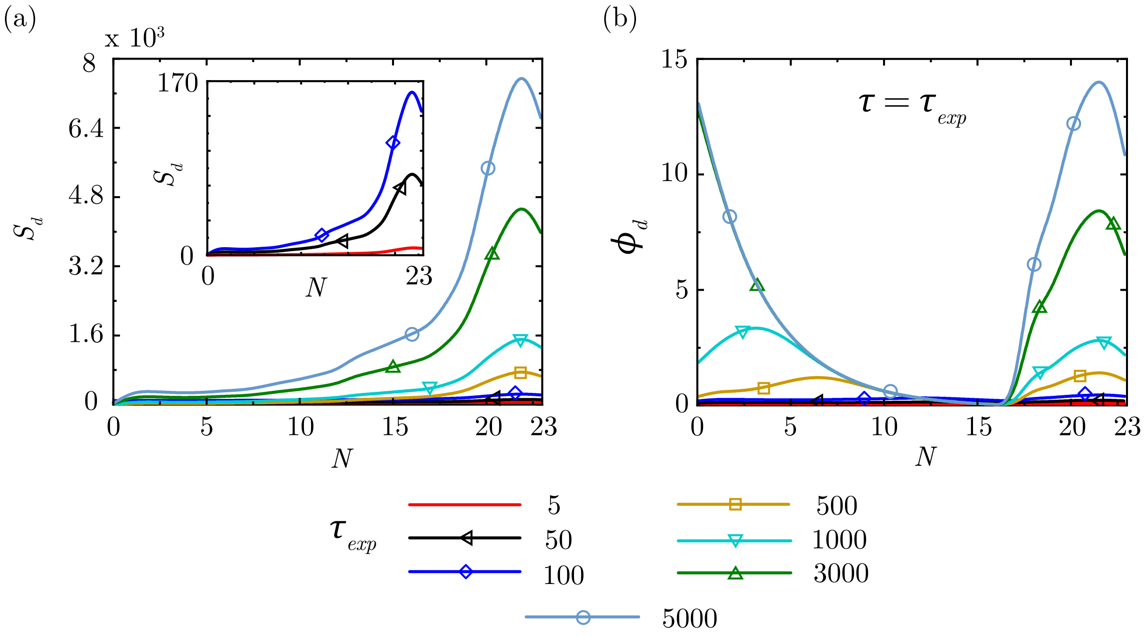

FIG. F. (a) Total aerosol deposition ($S_d = L'_D \phi_a$) within the lung for different τ_{exp}. Deposition for $\tau_{exp} = 5 - 100$ is additionally shown as inset to ensure proper readability (b) Drug concentration (ϕ_d) within the lungs for different τ_{exp} at the end of exposure i.e. at $\tau = \tau_{exp}$. The results are shown for $Pe_a = 2.85 \times 10^{10}$, $Pe_d = 4.56 \times 10^7$, $St_a = 0.0095$, $St_m = 359.7122$.

Fig Fa shows the total amount of aerosols deposited in the airway mucus for various τ_{exp} considered in this analysis. It can be observed that while the deposition pattern within the lungs remain almost identical, the magnitude of deposition increases as τ_{exp} become longer. The increase in deposition with exposure time is linear (see *Fig 4D* in the main manuscript). This observation can be used to estimate the dose of drugs that is delivered to a specific lung region over a particular period of time.

For example, pressurised meter-dose inhalers deliver 100 μg of salbutamol per puff and it usually takes 20-40 puffs to reverse the effects of bronchoconstriction[14]. Majority of the inhaled aerosolised drugs are deposited in the mouth and the pharynx, and only approximately 10% of the inhaled aerosolised drugs reach the trachea for further inhalation. A maximum of 28% of the aerosols that reach the trachea has been observed to reach the deep lung (corresponding aerosol size of 3 μm). Considering the above parameters, it is estimated that only 2.8 μg per puff i.e. 2.8% of inhaled drugs is able to reach the deep lung under

normal breathing conditions. Thus, for 40 puffs of inhaler, the total drug dose reaching the deep lung would be 112 μg. Assuming the entire drug dose deposited in the deep lung to be passed on to the blood circulation, the estimated drug concentration in blood would be 42.26 ng/ml considering the blood volume in children to be 2650 ml. Salbutamol concentration of $20-40$ ng/ml in blood is considered adequate for reversing bronchoconstriction in children[14]. Detailed calculation for the above estimation is as follows -

$$
\begin{aligned}
\textit{Dose per puff} &= 100\mu\text{g} \\
\textit{Dose per puff reaching the trachea} &= \textit{Dose per puff} \times \textit{Fraction of inhaled drugs reaching the trachea} \\
&= 100\mu\text{g} \times 10\% \\
\textit{Dose per puff reaching deep lung} &= \textit{Dose per puff reaching trachea}\times \\
&\textit{Fraction of inhaled drugs at trachea reaching deep lung} \\
&= 100\mu\text{g} \times 10\% \times 28\% \\
&= 2.8\mu\text{g} \\
\textit{Total dose reaching deep lung} &= \textit{Dose per puff reaching deep lung} \times \textit{Number of puffs} \\
&= 2.8\mu\text{g} \times 40 \\
&= 112\mu\text{g} \\
\textit{Drug concentration in blood} &= \textit{Total dose reaching deep lung}/\textit{Total blood volume} \\
&= 112\mu\text{g}/2650 \text{ ml} \\
&= 42.26 \text{ ng/ml}
\end{aligned}
$$

Similar calculations can be carried out for other combination of the pertinent parameters. The computational model can, thus, be utilised to estimate drug deposition and also to suggest ways to improve the drug delivery to the deep lung. Although the magnitude of drug deposition and drug concentration after a certain exposure duration can be determined by such extrapolations, it needs to be noted that this method is not a substitute for detailed simulations. Detailed simulations are still needed for drug retention calculation. The deposition characteristics can also change with variation in any one of the relevant parameters. Also, this knowledge does not provide information about the fraction of the inhaled aerosols that are deposited in the deep lungs. These informations can only be obtained from detailed simulations.

Fig Fb shows the drug concentration within the lungs for various τ_{exp} at the end of respective exposures. As expected, the drug concentration also increases due to larger aerosol deposition. In the upper airways ($N < 18$), mucuociliary transport clearance occurs simultaneously with aerosol deposition and as such, the effective drug concentration is the resultant of drug transport due to the deposition and clearance mechanisms. While drug concentration increases in these generations due to higher aerosol deposition, continuous mucus transport clears the drugs from these generations towards the 0th generation and as a consequence, drug accumulates in the first few generations (leading to much higher ϕ_d) before being washed out of the lung.

REFERENCES

[1]E. R. Weibel, A. F. Cournand, and D. W. Richards, *Morphometry of the human lung*, Vol. 1 (Springer, 1963).

[2]C. Karamaoun, B. Sobac, B. Mauroy, A. Van Muylem, and B. Haut, "New insights into the mechanisms controlling the bronchial mucus balance," PloS one **13**, e0199319 (2018).

[3]S. G. K. Devi, *Aerosol Deposition Studies in Human Lung - Towards Personalized Medicine*, Ph.D. thesis, IIT Madras, India (2018).

[4]D. B. Taulbee and C. Yu, "A theory of aerosol deposition in the human respiratory tract," Journal of Applied Physiology **38**, 77–85 (1975).

[5]S. K. Devi, M. V. Panchagnula, and M. Alladi, "Designing aerosol size distribution to minimize inter-subject variability of alveolar deposition," Journal of Aerosol Science **101**, 144–155 (2016).

[6]C. Darquenne and M. Paiva, "One-dimensional simulation of aerosol transport and deposition in the human lung," Journal of applied physiology **77**, 2889–2898 (1994).

[7]C. Mitsakou, C. Helmis, and C. Housiadas, "Eulerian modelling of lung deposition with sectional representation of aerosol dynamics," Journal of Aerosol Science **36**, 75–94 (2005).

[8]A. Chakravarty, N. A. Patankar, and M. V. Panchagnula, "Aerosol transport in a breathing alveolus," Physics of Fluids **31**, 121901 (2019).

[9]W. Hofmann, "Modelling inhaled particle deposition in the human lung—a review," Journal of Aerosol Science **42**, 693–724 (2011).

[10]H.-C. Yeh and G. Schum, "Models of human lung airways and their application to inhaled

particle deposition," Bulletin of mathematical biology **42**, 461–480 (1980).

[11] J. Heyder, J. Gebhart, G. Rudolf, C. F. Schiller, and W. Stahlhofen, "Deposition of particles in the human respiratory tract in the size range 0.005-15 μm," Journal of aerosol science **17**, 811–825 (1986).

[12] J. Sznitman, "Respiratory microflows in the pulmonary acinus," Journal of biomechanics **46**, 284–298 (2013).

[13] J.-I. Choi and C. S. Kim, "Mathematical analysis of particle deposition in human lungs: an improved single path transport model," Inhalation toxicology **19**, 925–939 (2007).

[14] W. Sellers, "Inhaled and intravenous treatment in acute severe and life-threatening asthma," British journal of anaesthesia **110**, 183–190 (2013).
